# Lucio’s phenomenon, a mutilating manifestation of leprosy

**DOI:** 10.1590/0037-8682-0874-2020

**Published:** 2021-03-22

**Authors:** Eduardo Vinicius Mendes Roncada, Isabella Andrade Marques, Marilda Aparecida Milanez Morgado de Abreu

**Affiliations:** 1 Universidade do Oeste Paulista, Hospital Regional de Presidente Prudente, Serviço de Dermatologia, Presidente Prudente, SP, Brasil.; 2 Universidade do Oeste Paulista, Graduação em Medicina, Presidente Prudente, SP, Brasil.

**FIGURE 1:**
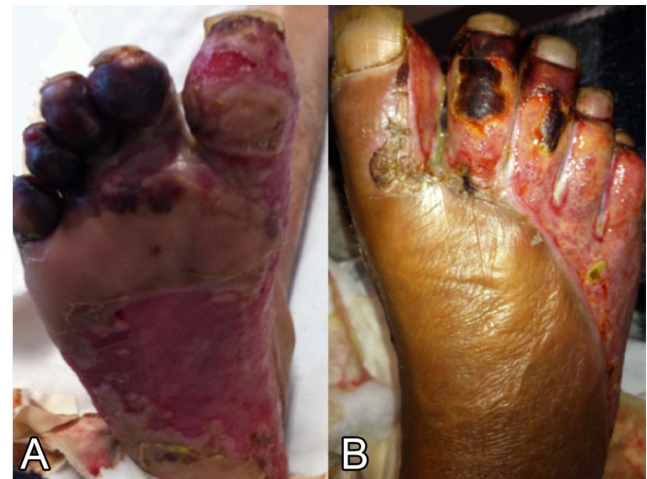
**(A)** Plantar region of the right foot with ulcerations and areas of necrosis on the toes at the first day of hospitalization. (**B)** Dorsal region of the right foot with areas of necrosis.

**FIGURE 2:**
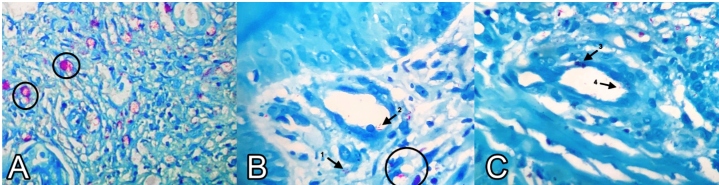
**(A)** Ziehl-Neelsen stain (40×) in this image; the Hansen’s bacilli are red stained with fuchsin, and they are isolated or bounded together forming the globi (black circles) in the macrophage’s cytoplasm, designated as Virchow’s cells; **(B)** Ziehl-Neelsen stain (100×). In the upper dermis, the presence of numerous Hansen’s bacilli was observed, isolated (arrow 1) and in clusters (black circle). Another important characteristic such as the presence of Hansen’s bacilli invading the vessel (arrow 2) can be seen; **(C)** Ziehl-Neelsen stain (400×). The presence of Hansen’s bacilli through the dermis and surrounding the blood vessel, invading the vascular wall (arrow 3) and in the vascular lumen (arrow 4).

**FIGURE 3:**
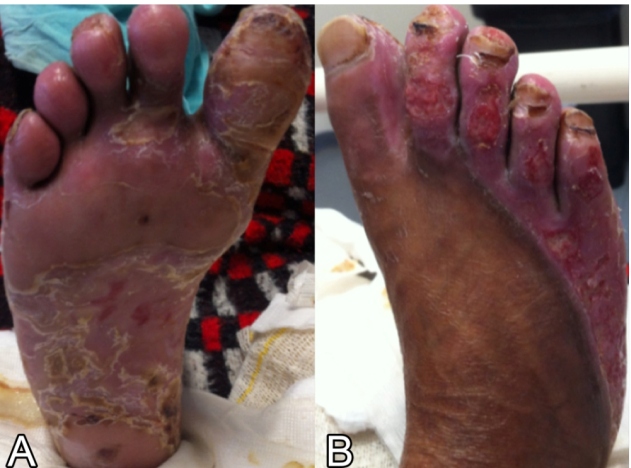
(A) Plantar region of the right foot with significant improvement. (B) Dorsal region of the right foot with significant improvement 20 days after starting the specific treatment.

A 44-year-old man was referred to our hospital with purple spots on his skin seven days prior. Dermatological examination revealed macules, erythematous violaceous papules, hemorrhagic blisters, and ulcerations with a clean background in the legs, feets, and hands were observed. No comorbidities or use of medications was noted. A similar episode occurred two years ago, in which the fifth left pododactyl was amputated. During hospitalization, he developed necrotic areas in his right toes (Figure 1). Laboratory test results were as follows: anti-cardiolipin IgG, 24.7 (positive: >20 GLP); IgM, 150 (positive: >20 MPL), antinuclear factor positive 1:160; and mixed standard, fine dotted nuclear, homogeneous nucleolar, and spindle-type mitotic patterns were observed. Histopathological examination revealed diffuse histiocytic infiltrate; a foamy appearance; several alcohol-acid-resistant bacilli in Ziehl-Neelsen's stain, sometimes in the wall and inside the vessel; and presence of erythrodiapedesis and eosinophils (vasculitis) (Figure 2). However, the other tests did not change. The diagnosis was lepromatous leprosy, suggesting Lucio’s phenomenon (LF). After starting the specific treatment, there was a significant improvement in the lesions and elimination of necrotic areas in three weeks (Figure 3). The patient was discharged from the hospital and was followed up as an outpatient.

LF is a rare manifestation of lepromatous leprosy^1^ ^,^ ^2^. It is clinically characterized by multiple painful erythematous violaceous macules and hemorrhagic blisters, which evolve into necrotic and ulcerated lesions^2^. Generally, this condition affects the upper and lower extremities. Patients who were diagnosed late can suffer from complications such as sepsis, amputations, and death from blood clotting disorders^3^.
